# The Promising Therapeutic Approaches for Radiation-Induced Pulmonary Fibrosis: Targeting Radiation-Induced Mesenchymal Transition of Alveolar Type II Epithelial Cells

**DOI:** 10.3390/ijms232315014

**Published:** 2022-11-30

**Authors:** Ping Wang, Ziyan Yan, Ping-Kun Zhou, Yongqing Gu

**Affiliations:** Beijing Key Laboratory for Radiobiology, Beijing Institute of Radiation Medicine, AMMS, Beijing 100850, China

**Keywords:** radiation-induced pulmonary fibrosis, epithelial–mesenchymal transition, alveolar type II epithelial cells

## Abstract

Radiation-induced pulmonary fibrosis (RIPF) is a common consequence of radiation for thoracic tumors, and is accompanied by gradual and irreversible organ failure. This severely reduces the survival rate of cancer patients, due to the serious side effects and lack of clinically effective drugs and methods. Radiation-induced pulmonary fibrosis is a dynamic process involving many complicated and varied mechanisms, of which alveolar type II epithelial (AT2) cells are one of the primary target cells, and the epithelial–mesenchymal transition (EMT) of AT2 cells is very relevant in the clinical search for effective targets. Therefore, this review summarizes several important signaling pathways that can induce EMT in AT2 cells, and searches for molecular targets with potential effects on RIPF among them, in order to provide effective therapeutic tools for the clinical prevention and treatment of RIPF.

## 1. Introduction

Local normal tissues are also exposed to ionizing radiation during radiation therapy for thoracic cancers such as breast cancer, lung cancer, and esophageal cancer, resulting in serious radiotherapy side effects, including radiation-induced lung injury (RILI). We can divide the process into early acute radiation pneumonitis and late chronic radiation-induced pulmonary fibrosis (RIPF) [1,2,3]. Progressive and irreversible destruction of lung tissue and deterioration of lung function characterizes radiation-induced pulmonary fibrosis, with clinical manifestations of dyspnea, interstitial effusion, and even respiratory insufficiency [3,4]. According to statistics, the incidence of RIPF due to radiotherapy varies widely, but can be as high as 50% [3,4,5], thus severely limiting the application of radiotherapy in the treatment of clinical thoracic tumors. Because of its complex pathogenesis and the lack of effective therapeutic targets, there are a lack of effective treatment strategies in clinical practice [4,5]. General studies have shown that in RIPF, activated myofibroblasts secrete a large number of extracellular matrix (ECM) components in the inflammatory tissues, which greatly favors pulmonary fibrosis [1,3,6,7,8]. Moreover, irradiated alveolar type II epithelial (AT2) cells can acquire a mesenchymal phenotype through epithelial–mesenchymal transition (EMT) and serve as the main source of myofibroblasts in the RIPF [3,6,7]. Therefore, exploring the specific molecular pathway of radiation-induced EMT of AT2 cells is necessary to find new molecular targets and intervention methods for the treatment of RIPF.

## 2. Radiation-Induced EMT Promotes RIPF

### 2.1. RIPF

The pathological process of RIPF is mostly characterized by infiltration of inflammatory cells in the lower airways, damage to alveolar epithelial cells and vascular endothelial cells, and the proliferation of fibroblasts and myofibroblasts which secrete large amounts of extracellular matrix proteins (ECM) and collagen—ultimately damaging pulmonary structures [6,9]. Due to its complex pathogeny and poor prognosis, there is no effective treatment for RIPF. Therefore, the clinical approach to the treatment of RIPF focuses on prevention, which means avoiding and minimizing the exposure of normal lung tissue when giving high doses of radiation to the tumor. Until now, FDA-approved drugs for the treatment of pulmonary fibrosis, such as Pirfenidone and Nintedanib, can only alleviate the progression of pulmonary fibrosis to a certain extent, and are associated with serious side effects such as nephrotoxicity [10,11]. Therefore, the search for therapeutic targets of RIPF is of great importance for the clinical development of new treatments.

### 2.2. Radiation-Induced EMT

Radiation-induced EMT of alveolar epithelial cells has been considered to be an important source of pulmonary fibroblasts, which are responsible for the expression and secretion of ECM during RIPF [1]. Additionally, EMT is a complex process in which radiation activates various signaling molecules to trigger irradiation-induced EMT by enhancing reactive oxygen species (ROS) [1,7,9]. Then, the epithelial cells lose intercellular adhesion and gradually transform into mesenchymal cells, which occurs primarily during tissue injury, wound healing, organic fibrosis, and tumor metastasis [12,13,14,15,16,17]. Type I EMT participates in embryo implantation and organ development, Type II EMT favors tissue regeneration and fibrosis of organs, and type III EMT is involved in tumor metastasis [18,19,20,21]. The transcription regulation and gene expression models of epithelial cells are modified, in which transcription factors such as Snail, Slug, ZEB1, Twist, and TCF/LEF are implicated in cell reprogramming as key regulators of EMT, leading to decreased epithelial markers E-cadherin and ZO-1 as well as increased mesenchymal markers N-cadherin, vimentin, and α-SMA [12,13,14,17,18,19]. In addition, epithelial cells move from oval cells to fibroblasts, which then differentiate into myofibroblasts secreting extracellular matrix [13,14,15,16,17], including collagen (particularly types I and III), glycoproteins, and proteoglycans (fibronectin, laminin), which exacerbate fibrosis in tissue organs [12,13,19].

**Figure 1:**
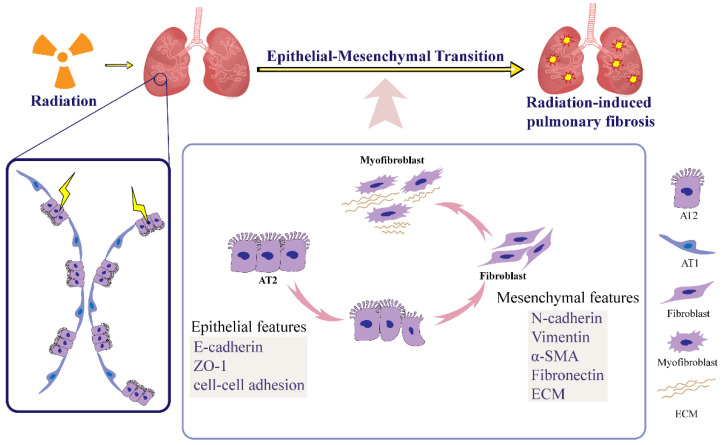
Radiation-induced EMT. Ionizing radiation induces epithelial–mesenchymal transition in AT2 cells, which lose their tight intercellular junctions and gradually lose their epithelial phenotype, eventually allowing them to acquire a mesenchymal phenotype and differentiate into fibroblasts. Thereafter, fibroblasts proliferate and activate in large numbers, further differentiating into myofibroblasts, which secrete large amounts of extracellular matrix components, leading to excessive deposition of extracellular matrix and promoting the development of RIPF. (AT1-alveolar type I epithelial, AT2-alveolar type II epithelial, ECM-extracellular matrix, ZO-1-zonula occludens-1).

Alveolar type II epithelial (AT2) cells function as progenitors or stem cells that participate in the repair of pulmonary injury through self-renewal and differentiation into alveolar type I epithelial cells (AT1) [22,23,24]. In idiopathic pulmonary fibrosis (IPF), AT2 cells can acquire a mesenchymal phenotype through EMT, followed by differentiation into myofibroblasts to produce large amounts of ECM, leading to pulmonary fibrosis [6,25,26,27]. The EMT of AT2 cells leads to the exhaustion of alveolar epithelial stem cells, which reduces the repair capacity of the alveolar epithelium and speeds up pulmonary fibrosis in mice [28]. One study showed that in mouse models of IPF with only alveolar epithelial cells expressing β-gal, cells with increased vimentin were nearly always positive β-gal, suggesting that alveolar epithelial EMT is an important source of fibroblasts in pulmonary fibrosis [29]. Similarly, AT2 cells are considered to be part of an extremely important biological process during the RIPF (Figure 1) [6,9,30]. In studies of radiation-induced pulmonary fibrosis, the radiation dose at 6 Gy significantly inhibited the expression of E-cadherin and promoted the expression of vimentin in AT2 cells [9,25,26]. More powerfully, S1PR3 gene deficiency in the lung significantly ameliorated RIPF in recombinant adeno-associated virus-mediated S1PR3 (AAV-S1PR3) gene-deficient mice, as evidenced by reduced collagen deposition and elimination of α-SMA; additionally, irradiation-induced EMT and fibrosis were significantly suppressed in S1PR3-deficient alveolar epithelial cells [30].

During the development of RIPF, triggering ionizing radiation (IR)-induced EMT in AT2 cells requires appropriate extracellular signals in addition to IR-induced ROS, which include ECM components (integrins, MMPs, collagen) and several polypeptide growth factors such as TGF-β, Connective tissue growth factor (CTGF), Platelet-derived growth factor (PDGF), hepatocyte growth factor (HGF), fibroblast growth factor (FGF), Epidermal Growth Factor (EGF), and vascular endothelial growth factor (VEGF) that can interact with cognate receptors (tyrosine kinase receptors or serine/threonine kinase receptors) outside the cell membrane [1,13,17,22,23]. In addition, many complex and crosstalk intracellular signaling pathways are involved in the occurrence of EMT in AT2 cells. Recent studies suggest that ROS, Polypeptide growth factor, NF-κB, PI3K/AKT, epigenetic factors (MicroRNAs), Wnt/β-catenin, and Notch are all triggers involved in EMT in radiation-induced pulmonary fibrosis [1,2,3,4,5,6,7,8,9,24]. Although there are many studies involving the molecular mechanisms of RIPF, these studies have not yet been effectively translated into clinical applications.

## 3. Signaling Pathway Involved in EMT of AT2 Cell in RIPF

### 3.1. TGF-β Signaling Pathway

**Figure 2:**
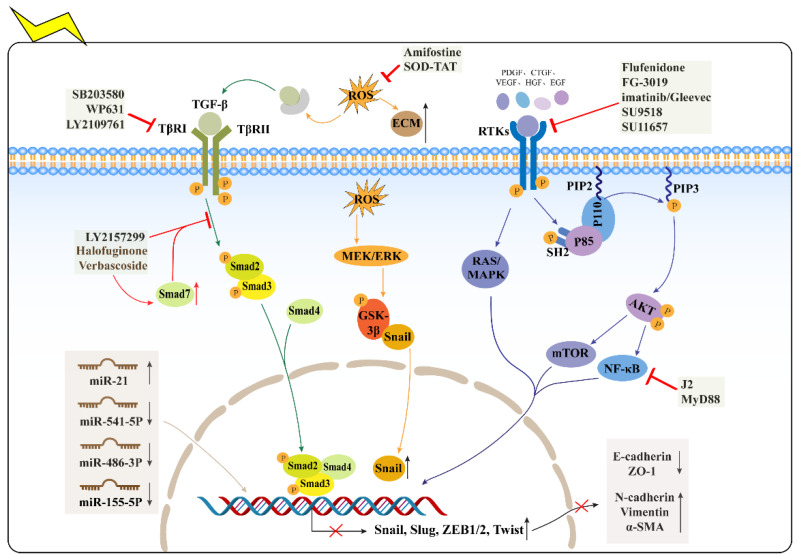
Signaling pathways and targets of EMT, and its therapeutic approach in RIPF. ROS, TGF-β signaling pathway, growth factors and MicroRNA are promising targets for therapeutic and developmental applications in RIPF, and their targeted inhibitors have potent inhibitory effects on IR-induced EMT in AT2 cells, respectively, inhibiting further development of RIPF. (TGF-β- Transforming growth factor β, TβR1-TGF-β type I receptor, TβR2-TGF-β type II receptor, ROS- Reactive oxygen species, RTKs- receptor tyrosine kinases, PIP2-phosphatidylinositol-4,5-biphosphate, PIP3-phosphatidylinositol triphosphate, α-SMA-α-smooth muscle actin). All arrows indicate the transduction process and protein changes in the EMT signaling pathway, and the red crosses indicate that the above inhibitors can ultimately suppress the change of EMT-related marker proteins by inhibiting the elevation of transcription factors, ultimately inhibiting IR-induced EMT in AT2.

Transforming growth factor β (TGF-β) is a pleiotropic cytokine with three distinct isoforms, including TGF-β1, TGF-β2, and TGF-β3, that regulate cellular proliferation, differentiation, EMT, and immune regulation [3,25,26,27]. TGF-β is generally present in the ECM as a potential complex and can be separated from the potential complex to bind to its receptors for action, including the TGF-β type I receptor (TβR1) and the TGF-β type II receptor (TβR2) [3,25,26]. It has been noted that IR-induced ROS can promote the separation of TGF-β from the latent complex (Figure 2), so that activated TGF-β can bind to TGF-β type II receptor (TβR2) on the AT2 cell membrane, finally inducing ECM production and myofibroblast proliferation to promote RIPF by triggering the IR-induced EMT signal transduction pathway [22,28,29,30]. Additionally, in the TGF-β/Smad signaling pathway, TGF-β attaches to TβR2 to recruit TβR1 into the plasmatic membrane to form a heterotrimer. The heterotrimer phosphorylates Smad2/3 proteins (a process inhibited by Smad7) to form a complex with Smad4, which is subsequently transferred to the nucleus to interact with transcription factors to regulate transcription, translation, and protein synthesis of target genes [25,26]. In addition, the non-Smad pathway is associated with TGFβ-induced EMT through activation of multiple signaling pathways (PI3K-AKT, ERK-MAPK, p38-MAPK, JNK, RhoA, and NF-κB) [2,9,31].

The TGFβ-induced EMT in AT2 cells plays a key role in the development of RIPF. As the critical cytokine in pulmonary fibrosis, radiation-activated TGF-β triggers the EMT in AT2 cells (Figure 2), which increases mesenchymal genes N-cadherin and vimentin, as well as inhibiting epithelial genes ZO-1 and E-cadherin; it also transforms the morphology of epithelial cells into mesenchymal cells to produce a large amount of ECM [27,32]. Activated TGF-β carried by adenovirus may promote fibrosis in rodent models [6], and blocking TGF-β may attenuate the development of RIPF [3]. In addition, alveolar cells can be a source of TGF-β in pulmonary fibrosis. Similarly, irradiated mouse alveolar epithelial cells may release large amounts of chemokine to recruit M2 macrophages, and TGF-β secreted by M2 macrophages can induce EMT in MLE-12 to increase the expression of N-cadherin and promote the progression of RIPF [6]. In conclusion, the TGF-β pathway is the most promising therapeutic target for RIPF. Given that TGF-β is activated in ECM before it can bind to receptors on the AT2 cell membrane, and then regulate transcription factors in a Smad and non-Smad-dependent manner to promote the mesenchymal phenotype, clinical studies have mainly focused on inhibiting the synthesis and activation of TGF-β and blocking the signaling pathway.

### 3.2. Tyrosine Kinases Pathway

In RIPF, damaged alveolar epithelial cells, resident macrophages, and endothelial cells all secrete a variety of proinflammatory and profibrotic growth factors [1,33]. Previous studies have confirmed that the occurrence of chronic lung diseases is related to the overexpression of the growth factor ligands, or the increased expression of the receptor tyrosine kinases (RTKs) [34]. When related RTKs interact with polypeptide growth factor ligands including FGF, VEGF, EGF, HGF, and PDGF (Figure 2), these ligands can promote the dimerization of RTKs and autophosphorylation of their tyrosine residues, which can serve as docking sites for SH2 domains to activate related downstream signaling pathways such as RAS/MAPK and PI3K/AKT pathways, and initiate IR-induced EMT [22,23,35]. For example, many studies have shown that PDGF may play a role in EMT by promoting β-catenin translated into the nucleus in cooperation with TGF-β [36]. The expression of PDGF is up-regulated in the lung tissue of C57BL/6 mice with radiation-induced lung injury [37,38], and blocking PDGF by the inhibitors of RTKs can reduce the development of RIPF [3,38,39], which suggests that the PDGF–PDGFR system is a very promising potential target for the treatment of RIPF. Connective tissue growth factor (CTGF) is reported to be a TGF-β-mediated pro-fibrotic growth factor that promotes collagen synthesis and ECM deposition [40,41], thus is considered a potential molecular target to alleviate RIPF.

### 3.3. PI3K/AKT Signaling Pathway

PI3K is an intracellular phosphatidylinositol kinase that has three categories. Class I PI3K is a dimer consisting of catalytic subunit p110 and regulatory subunit p85 [42,43,44,45,46,47]. The activation signal for PI3K is mainly a variety of growth factors/signal transduction complexes, such as FGF, VEGF, EGF, HGF, and insulin [48,49]. In response to growth factor stimulation, the p85 regulatory subunit binds to the phosphotyrosine residues of the growth factor receptor, via its SH2 structural domain and the phosphotyrosine motif on the RTKs (Figure 2), recruiting the p110 catalytic subunit to reach the cell membrane and phosphorylate PIP2 [43,48]. Activated PI3K catalyzes PIP2 phosphorylation into PIP3 (a process inhibited by PTEN), which activates the protein kinase B (PKB or AKT) [49,50,51]. When phosphorylated at the Ser473 and Thr308 sites, AKT can control downstream pathways, including mTOR, MAPK, glycolysis, cell cycle, and apoptosis [42,49].

AT2 cells, in several studies, can promote the development of RIPF through PI3K/AKT signaling pathway-induced EMT. Previous studies have indicated that Re-Du-Ning (RDN) therapy may attenuate RILI in mice via the PI3K/AKT signaling pathway to suppress EMT in alveolar epithelial cells [52]. Additionally, TBK1, as a potential therapy target for RIPF, can directly activate the AKT signaling pathway to promote EMT in alveolar epithelial cells. Moreover, the PI3K/AKT signaling pathway can be activated in bleomycin-induced pulmonary fibrosis (the expression of p-PI3K and p-AKT are increasing) [53,54,55,56,57]. In DEC-deficient mice and cells, DEC deficiency improves idiopathic pulmonary fibrosis by inhibiting the PI3K/AKT/GSK-3β/β-Catenin signaling pathway to suppress EMT in AT2 cells [58]. Treatment with low molecular weight fucose (LMWF) relieves pulmonary fibrosis and EMT by inhibiting the PI3K/AKT signaling pathway, in vivo and in vitro [59]. In particular, the PI3K/AKT signaling pathway is a key target for treating COVID-19-related pulmonary fibrosis [57]. It is thus clear that since AKT downstream signaling triggered by PI3K activation induces significant EMT in AT2 cells, selecting PI3K as a new potential target and inhibiting its activation is more comprehensive and efficient for the treatment of RIPF.

### 3.4. Epigenetic Factors (MicroRNAs)

Epigenetics refers to the production of heritable phenotypic changes without altering the DNA sequence by mechanisms including DNA methylation, histone modifications and non-coding RNAs [60]. Epigenetics has been shown to play a key role in immune, cancer, endocrine and psychiatric disorders, and epigenetic aberrations can be reversed by drugs [61]. Recent studies have pointed out that epigenetics (such as DNA methylation, m6A modification of RNA, histone modification and the binding of non-coding RNAs to mRNA) can regulate the development of RIPF by promoting or inhibiting the expression of downstream target genes to regulate the activation of EMT-related signaling pathways, and promote RIPF [62,63,64]. Among these, non-coding RNAs are very promising biomarkers to regulate RIPF in epigenetics, which mainly include microRNA (miRNA), long noncoding RNA (lncRNA), circular RNA (circRNA) [60]. Additionally, our findings suggest a clear role for MicroRNAs in mouse RIPF.

MicroRNAs are a class of small non-coding RNAs containing 19–25 nucleotides that can play an important role in the post-translational gene regulation and expression, through gene silence and epigenetics [12,16]. As non-coding genes, MicroRNAs can regulate the expression of major transcription factors in EMT such as Snail, ZEB1/2, Slug or target genes (Figure 2), promoting or inhibiting the progression of EMT [12]. MicroRNA dysregulation is closely associated with initiating EMT in AT2 cells. Current studies have reported that abnormal expression of miR-21 exists in multiple aspects of pulmonary fibrosis, including EMT of alveolar epithelial cells and differentiation of fibroblasts into myofibroblasts [65,66]. Therefore, miR-21 is expected to become a new candidate MicroRNA for intervention in radiation-induced pulmonary fibrosis. In addition to miR-21, our laboratory found that the ectopic expression of miR-155-5p in the lungs of C57BL/6 mice, inverted IR-induced EMT in AT2 cells and attenuated RIPF in mice by downregulating GSK-3β [67]. Similarly, IR could significantly induce Snail and Slug expression in AT2 cells by, respectively, inhibiting miR-486-3p and miR-541-5p (Figure 2), triggering IR-induced EMT and inducing the development of RIPF [68,69]. Through the intervention of the mentioned MicroRNAs, it was found that the reversal effect of IR-induced EMT was very obvious, and the pathological results of RIPF in mice were significantly improved; this suggested to us that targeting miRNAs may be an effective strategy to improve the current treatment dilemma of RIPF.

## 4. Therapy Strategy of RIPF by Targeting EMT

### 4.1. Current Clinical Situation of Pulmonary Fibrosis

Currently, the drugs approved by the FDA for the treatment of pulmonary fibrosis are Nintedanib and Pirfenidone, both of which target the activation and proliferation of fibroblasts, thereby acting to inhibit the progression of pulmonary fibrosis. However, as research has progressed, numerous in vivo studies have shown that Nintedanib and Pirfenidone can also target the epithelial–mesenchymal transition of AT2 cells and mitigate the progression of lung fibrosis [70,71,72]. In addition, FG-3019, a human-derived CTGF monoclonal antibody, inhibits the conversion of epithelial cells into fibroblasts via EMT to inhibit pulmonary fibrosis [41]. FG-3019 has now completed Phase I and Phase II clinical trials and is currently undergoing Phase III clinical studies. In a phase II randomized placebo-controlled trial, administration of FG-3019 significantly reduced the decline in pulmonary function compared to placebo, and quantitative pulmonary fibrosis HRCT scores showed that FG-3019 slowed progression in patients with pulmonary fibrosis, was safe and well tolerated, and significantly improved patients’ quality of life [73,74]. Moreover, pulmonary fibrosis is not only one of the sequelae of radiotherapy for chest tumors, but also one of the pathological features of COVID-19 patients. The current national medical program-recommended Chinese medical formula for COVID-19 patients is Qimai Feiluoping decoction, which can significantly improve pulmonary function and reduce clinical symptoms in the clinical phase [75]. Its potential mechanisms of action have been identified through network pharmacology predictions, including inhibition of the TGF-β/Smad3 pathway to inhibit TGF-β1-induced AT2 cell proliferation, and inhibition of EMT to alleviate pulmonary fibrosis [75,76].

However, there are currently limited clinically available drugs for the treatment of pulmonary fibrosis, and clinical data on targeted EMT for the treatment of patients with pulmonary fibrosis are also scarce. We therefore note that many of the anti-tumor drugs currently used in clinical patients are targeted to EMT [77,78]. Galunisertib, the first TβR1 inhibitor to be advanced to clinical trials, inhibits TGF-β pathway-induced EMT in tumor cells. Additionally, clinical data showed lower protein expression levels of p-Smad2 in peripheral blood mononuclear cells of patients receiving the clinically beneficial Galunisertib [79,80]. Vactosertib is also a novel TβR1 inhibitor, currently under development in clinical trials, that inhibits the phosphorylation of Smad2 and Smad3 proteins, thereby suppressing EMT in tumor cells, and has been shown to be safe and well tolerated in phase I clinical studies [80,81,82]. In addition, Gefitinib (an EGFR-tyrosine kinase inhibitor) is among the anti-tumor drugs involved in the inhibition of the EMT process in tumor cells, with good results in clinical studies so far [83,84]. Although all of these clinical agents mentioned above are targeting EMT of tumor cells in cancer patients, investigating their effects on pulmonary fibrosis in vivo is an effective way of providing us with the ability to rapidly develop new drug candidates for the treatment of pulmonary fibrosis, due to their reliable safety and tolerability.

### 4.2. Pre-Clinical Treatment of RIPF

#### 4.2.1. Targeting TGF-β Signaling Pathway

In RIPF, activation of the TGF-β/Smad signaling pathway is a crucial step. Nowadays, therapeutic strategies in clinical studies regard TGF-β as a potential target that can be divided into three types (Table 1): inhibits latent TGF-β synthesis (antisense molecules), inhibits the activation of TGF-β (neutralizing antibody), and inhibits downstream gene transduction (receptor kinase inhibitor) [25].

For example, as a major TGF-β activator in the pulmonary system, direct inhibition or knockdown of integrin αvβ6 is a potential therapeutic target for RIPF [85]. In addition, removal of ROS also can inhibit the activation of the TGF-β signaling pathway to some extent, as ROS activates TGF-β. Additionally, studies show that Amifostine is currently used as a clinical antiradiation drug with an active metabolite as a ROS scavenger (Table 1) and is a potential drug for the treatment of radiation-induced pulmonary fibrosis [86,87]. To prevent ROS, the superoxide dismutase fusion of TAT (SOD-TAT) significantly ameliorated radiation-induced lung injury in mice [88].

As TβR1 inhibitors, LY2109761 [1,2,3,25,89] directly inhibited pro-fibrotic signaling and balanced the TGF-β/BMP signaling pathway in the RIPF, Galunisertib (LY2157299) [3,25,90] downregulated Smad2 phosphorylation in lung cancer, SB203580 and WP631 inhibited downstream signaling by highly specific inhibition of TβR1 [1,2,91], and SM16 [1,2,92] significantly reduced the extent of RIPF in rats. Furthermore, Smad7, a TGF-β pathway inhibitor, inhibits TGF-β signaling transduction by inhibiting blocking of Smad2/3 phosphorylation [26,27,93]. The natural products, Halofuginone and Verbascoside, can inhibit TGF-β pathway activity by inhibiting Smad2/3 phosphorylation and increasing Smad7 mRNA expression [26,94,95], which are potential RIPF protective molecules.

As mentioned above, there are a wide variety of inhibitors targeting the TGF-β pathway, including antitumor drugs that have entered clinical trials and gained FDA approval. The investigation of the pharmacological activities and molecular mechanisms of these drugs can facilitate the development of new clinical applications, and provide a more immediate and effective approach for the clinical treatment of tumor patients with RIPF induced by radiotherapy. Moreover, the TGF-β signaling pathway is a powerful molecular mechanism in IR-induced EMT, that can amplify the regulatory effects on EMT by activating signaling molecules from other pathways or by crosstalk with other signaling pathways. Therefore, the development of inhibitors of the TGF-β pathway is of great potential value.

#### 4.2.2. Targeting Growth Factors

Damaged AT2 cells, macrophages and endothelial cells secrete large amounts of pro-fibrotic growth factors during the EMT in AT2 cells, by regulating pathways such as the PI3K/AKT pathway [96,97,98]. Therefore, the growth factors have been considered as signaling molecules and targets associated with the development of RIPF (Table 1). Flufenidone (a novel anti-fibrotic agent) reduces cardiac and renal fibrosis by inhibiting CTGF expression [99]. Moreover, PDGF promotes idiopathic pulmonary fibrosis (IPF), bleomycin-induced pulmonary fibrosis, and RIPF [37,100]. Additionally, PDGF receptor tyrosine kinase inhibitors (imatinib/Gleevec, SU9518 and SU11657) significantly attenuated the progression of pulmonary fibrosis lesions and remodeled lung structure in animal models [38,39,101]. In addition, PDGF receptor tyrosine kinase inhibitors are currently in clinical trials [23]. It means that these inhibitors may have more obvious efficacy, selectivity and safety in the treatment of RIPF, and are expected to be candidate medicines for the prevention and treatment of RIPF in the future.

#### 4.2.3. Additional Targets

In addition, we found that Syndecan-2 can inhibit fibroblast differentiation and reduce pulmonary fibrosis in irradiated mice by downregulating PI3K/AKT pathway activity [102]. 2-Methoxyestradiol (2-ME) inhibits radiation-induced elevation of HIF-1α levels, reduces vascular collagen deposition, and inhibits the development of RIPF by inhibiting radiation-induced EndMT and EMT [103]. Additionally, the small molecule inhibitor J2 of heat shock protein-27 (HSP-27), a candidate target used in mouse models for the treatment of pulmonary fibrosis (Table 1), inhibits the development of RIPF by inhibiting IkBa-NFkB signaling after cross-linking HSP27 [104]. It is likely that MyD88 (a key intracellular adapter for TLR signaling) prevents pulmonary fibrosis by regulating NF-κB activation to attenuate RILI (Table 1) [105]. The SIK2 (salt-inducible kinase-2) inhibitor ARN-3236 was recently reported to attenuate bleomycin-induced pulmonary fibrosis in mice [106]. This gives us new candidate targets for a more comprehensive search for appropriate therapeutic approaches.

**Table 1:** Therapeutic strategies and inhibitors for radiation-induced pulmonary fibrosis.

Mechanism	Name	Structural Formula	Type	Target	Ref.
ROS	Amifostine	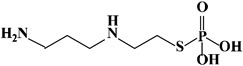	Organic thiophosphate	Scavenging free radicals	[86,87]
	SOD-TAT	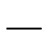	Recombinant protein	Oxidative damage	[88]
TGF-β signaling pathway	LY2109761	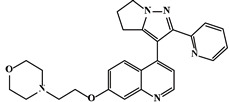	Quinoline derivatives	TβR1 inhibitor	[89]
	Galunisertib	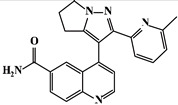	Pyrrolopyrazole	TβR1 inhibitor	[90]
	SB203580	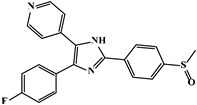	Imidazoles	TβR1 inhibitor	[91]
	WP631	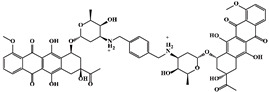	Bisintercalating anthracycline	TβR1 inhibitor	[91]
	SM16	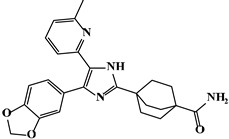	Antibody	TβR1 inhibitor	[92]
	Halofuginone	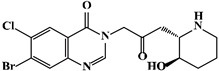	Quinazolinone alkaloid	Smad2/3 phosphorylation	[94]
	Verbascoside	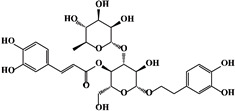	Glucosides	Smad2/3 phosphorylation Increasing Smad7	[95]
Tyrosine kinase pathway	Flufenidone	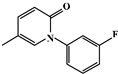	Anti-fibrotic drug	CTGF	[99]
	FG-3019	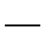	Recombinant antibody	CTGF	[41]
	Imatinib	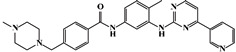	Antineoplastic agent	PDGF	[101]
	SU9518	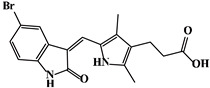	Small molecule	VEGF PDGF	[38,39]
Addition	2-ME	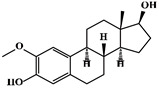	17β-hydroxy steroid	PI3K/AKT HIF-1α	[103]
	J2	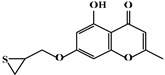	Small molecule	IkBa-NFkB HSP-27	[104]
	MyD88	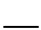	Recombinant protein	NF-κB activation	[105]

## 5. Conclusions

Early diagnosis and treatment of RILI in patients with thoracic tumors after radiotherapy is now agreed to be a very important treatment to prevent the development of RIPF. However, although precision radiotherapy can reduce RILI, it affects tumor outcomes and quality of survival, and therefore RIPF remains a pressing challenge for medical research to overcome. RIPF is a progressive disease that involves many complex molecular mechanisms, with various molecular pathways crosstalking each other and cooperatively contributing to the pathological process of RIPF. In addition to molecular targets that target the IR-induced EMT signaling pathway, there are also many drugs in development or in clinical trials that target other molecular targets, such as by inhibiting the recruitment of inflammatory cells or targeting the clearance of senescent cells [107,108,109]. Recently, steroids, diuretics, hormones, antioxidants and enzymes are used clinically to treat RIPF, but because these drugs are not symptomatic and lack specificity, their treatment is not very effective [110,111]. Therefore, further investigation of the molecular mechanisms involved in RIPF is essential to identify new clinical targets.

Currently, it is well established that epithelial cells convert into migratory and invasive mesenchymal cells during tumor development, but the precise role of EMT as a source of pulmonary fibroblasts has been challenged by an AT2 cell fate tracking study in a bleomycin-induced mouse model of pulmonary fibrosis [112]. One study found no evidence of transformation of labeled AT2 cells into myofibroblasts via EMT when using surfactant protein C-CreERT2 knock-in alleles to follow the fate of AT2 cells in vivo [112]. Another study also showed that minor alveolar epithelial cells can be transformed into fibrotic lesions via EMT in a TGF-α-induced pulmonary fibrosis model [113]. Therefore, some researchers argued that the genetic lineage tracing methods—used early to provide evidence of EMT involvement in proliferation of pulmonary fibroblasts in IPF, using β-galactosidase as a genetic marker—cannot rule out false-positive results [13]. Thus, the in vivo evidence that EMT can directly convert into fibroblasts is still somehow unclear during PF/IPF [114,115]. Similarly, there is also no in vivo evidence to support the notion that AT2 cells could directly contribute to myofibroblast population via EMT in RIPF, and lineage tracing studies should be performed to uncover the EMT role during RIPF. Although it is controversial as to whether pulmonary epithelial cells could be directly converted to myofibroblasts via EMT in pulmonary fibrosis, some studies suggested EMT could promote myofibroblast differentiation by secreting factors to promote pulmonary fibrosis [116]. Another study revealed that radiation-induced EMT in AT2 cells can alter the microenvironment by secreting cytokines to recruit macrophages, thereby promoting RIPF [6]. Therefore, it is essential to investigate the molecular mechanisms of EMT in RIPF, which will provide us with more potential targets to find effective strategies for RIPF.

In conclusion, we have summarized the targets in the major IR-induced EMT signaling pathways and systematically collated the inhibitors of the molecular targets involved in EMT (Table 1), with a view of finding more effective therapeutic strategies for the treatment of RIPF, and developing drugs with better efficacy and specificity.

## Data Availability

Not applicable.
